# Dynamics of large-scale neuronal networks of the human cortex functional connectivity

**DOI:** 10.1186/1471-2202-13-S1-P117

**Published:** 2012-07-16

**Authors:** Vesna Vuksanović, Philipp Hövel

**Affiliations:** 1 Technische Universität Berlin, Germany; 2 Bernstein Center for Computational Neuroscience Berlin, Germany; 3 Northeastern University, Boston, Massachusetts 02115, USA

## 

Spatio-temporally organized low-frequency fluctuations (<0.1 Hz) of blood-oxygen-level-dependent (BOLD) fMRI signal have been intensively investigated as a measure of functional connectivity (FC) between region pairs in the whole brain [1]. Resting state FC is commonly assumed to be shaped by the underlying anatomical connectivity (AC). Furthermore, it has been suggested that the strength, persistence, and spatial properties of FC are constrained by the large-scale anatomical structure of the cortex [2]. However, strong resting state FC is often observed between pairs of remote cortical regions, even without apparent direct anatomical connections [3]. Mechanisms generating resting state FC are largely unknown, and it has been contended that indirect connections, interregional distance, and collective effects governed by network properties of the cortex play significant role. In addition, some theoretical studies on large-scale brain networks demonstrated the importance of time delays in networks dynamics for the generation of resting state FC fluctuations [4,5]. To address these questions we investigate large-scale neural network model of human cortex FC. Our model is based on an empirically derived resting state FC network consisting of 64 region of interest (ROIs) (network nodes), which are chosen from all over the cortex. The ROIs are adapted from a study of functional segmentation of the brain cortex using high-model-order independent component analysis (ICA) [6]. There are 30 pairs of inter-hemispheric homologues, and 4 additional ROIs are chosen along the midline. The activity of each node is described by FitzHugh-Nagumo neurons. Network dynamics is modelled with different parameters for each node and different time delays to account for the finite signal propagation times between the nodes.
